# Supplementary material for: Severe and mild-moderate SARS-CoV-2 vaccinated patients show different frequencies of IFNγ-releasing cells: An exploratory study
Source: PLoS One. 2023 Feb 9;18(2):e0281444. doi: 10.1371/journal.pone.0281444 (PMC9910754; doi:10.1371/journal.pone.0281444)
Supplement: Attachment — Submitted filename: Response to Reviewers.docx [file pone.0281444.s001.docx]

**Reviewer #1**

The authors should read these published articles to deep discuss their findings:
- Coppeta L, Ferrari C, Somma G, Mazza A, D'Ancona U, Marcuccilli F, Grelli S, Aurilio MT, Pietroiusti A, Magrini A, Rizza S. Reduced Titers of Circulating Anti-SARS-CoV-2 Antibodies and Risk of COVID-19 Infection in Healthcare Workers during the Nine Months after Immunization with the BNT162b2 mRNA Vaccine. Vaccines (Basel). 2022 Jan 18;10(2):141. doi: 10.3390/vaccines10020141. PMID: 35214600; PMCID: PMC8879462.

- Coppeta L, Somma G, Ferrari C, Mazza A, Rizza S, Trabucco Aurilio M, Perrone S, Magrini A, Pietroiusti A. Persistence of Anti-S Titre among Healthcare Workers Vaccinated with BNT162b2 mRNA COVID-19. Vaccines (Basel). 2021 Aug 25;9(9):947. doi: 10.3390/vaccines9090947. PMID: 34579184; PMCID: PMC8472926.

- Sahin U., Muik A., Vogler I. BNT162b2 Induces SARS-CoV-2-Neutralising Antibodies and T Cells in Humans. Preprint. [(accessed on 11 December 2020)]. Available online: <https://www.medrxiv.org/content/10.1101/2020.12.09.20245175v1>

*We thank the Reviewer for his/her suggestion. We included and quoted the two papers by Coppeta et al. in the Discussion section, whereas we prefer to not include the paper by Sahin, since this is a non-peer reviewed paper published as a pre-print. Therefore, this manuscript may contain some flaws requiring the revision process before publication and to consider it as scientifically valid manuscript.*

**Reviewer #2**

Major issues:
1) Definition of study groups: the authors define the groups as hospitalized and non-hospitalized. However, there are several reasons for admission of COVID-19 patients beside their disease severity. I recommend changing the groups (throughout the paper, title, and conclusion) to severe, mild-moderate and healthy subjects.

*We thank the Reviewer for his/her suggestion. We have completely followed her/his suggestion and cohorts have been renamed as required.*

2) Study design: For proper validation of the results and the ability to implement them in future studies, several important issues must be addressed by the authors:
Methods:

- How were patients selected? Was it during their stay in the emergency department?

*We have now included in the revised version of the manuscript this information.*

- At which point in the disease course the blood samples were drawn – was it at admission to the COVID-19 department?

*As already mentioned in the manuscript: “After inclusion, demographic and clinical features were recorded. Furthermore, blood samples were collected in BD Vacutainer plasma tubes containing lithium heparin tubes (Becton Dickinson; Plymouth, UK).”*

- Did patients with severe disease received dexamethasone (or other steroids) for their disease prior to their inclusion in the study? If so – for how many days?

*No patients received steroids before study inclusion. This is now specified in the results section.*

Results:
- What is the mean time from the vaccines to study inclusion for each group?

*The reviewer is right, we missed this information that is relevant. We have now included it in Table 1. Since we assumed data as non-parametric, we have described the time (i.e. days) from last vaccine dose in median [interquartile range].*

- What is the mean time from the PCR and from the first COVID-related symptoms to study inclusion for each group?

*Thanks, we have now specified this in Materials and Methods. Patients were classified in the 3 cohorts at the time of swab result that corresponds to the inclusion time.*

- Were any patients with mild/moderate disease deteriorated after being included in the study?

*None of the included mild/moderate patients deteriorated to severe after study inclusion. This is now specified in the revised text.*

- Any significant comparison between two of the three groups should be addressed in Table 1. Currently the footnote of the table addresses this issue, but it is not mentioned in the table.

*We thank the Reviewer for her/his indication. There are no significant differences among populations and footnotes have been deleted accordingly.*

#3: discussion: While this section is properly written and presents the main findings, I believe the authors need to further discuss the following ideas:
- What is your theory for the basis of your results?
Do you think the low levels of IFNγ-releasing cells were present in these patients before being infected with COVID-19, which means it can be screened in the wider vaccinated population? On the other hand, do you think these patients had an inappropriate response to the infection (which reflects in low levels of IFNγ-releasing cells, and is also the basis for the study you cite in citation 12) and therefore have severe disease? If so, screening is relevant only among infected patients.
Based on your theory you should relate to previous studies, to the results of the healthy subjects (and their high levels of IFNγ-releasing cells) and to the possible implications in the general population.

*We thank the Reviewer for his/her comment. We have included in the manuscript (discussion) this concept that is trivial. However, it remains difficult to state if our findings are related to the infection or a weak response to the vaccine. At this regard, we have left open the possibility and the question to a specifically designed trial.*

- Previous studies describe both high and low levels of IFNγ among patients with severe disease, while this was not discussed by the authors in relation to their findings.

Following the Reviewer’s suggestion, we have included a paragraph in the discussion.

- Levels of IgG-S were previously shown not to correlate with disease severity and outcomes. The authors should address it to support their findings. In this regard, I recommend them to use the following paper which showed similar results for comparison: <https://doi.org/10.1371/journal.pone.0268050>

*We thank the Reviewer for her/his thoughtful suggestion that has been fully included in the discussion.*

- The age of hospitalized patients was substantially older (*p* value is not everything when it’s a small number of patients). The authors should address this issue as a possible confounder or limitation.

*Done, thanks.*

Minor issues:

– In the end of the results, you state “A diagnostic specificity of 100% was observed for s.f.c. > 81.2 x 106…”. If I understand correctly, a lower value than 81.2 is indicative for a severe disease not higher. If so it should be changed accordingly.

*In our study, diagnostic specificity refers to the IFN-γ ELISPOT's ability to correctly define patients with mild / moderate disease, which is 100% for s.f.c. greater than 81.2/PBMCs 10^6^.*

**Reviewer #3**

First, this is certainly not the first manuscript that analyze T cell response in patients with severe or mild or asymptomatic SARS-CoV-2 infection.

As such the introduction should acknowledge the work on T cell response published by other authors that have already showed presence of T cells not only in Covid-19 but also on asymptomatic individuals ( i.e Sekine, T. et al. Robust T Cell Immunity in Convalescent Individuals with Asymptomatic or Mild COVID-19. Cell 183, 158-168.e14 (2020), Le Bert, N. et al. Highly functional virus-specific cellular immune response in asymptomatic SARS-CoV-2 infection. J Exp Med 218, e20202617 (2021).). Furthermore, the abstract (and discussion) does not clarify that the analysis of Spike-specific T cell response was done after infection and not before the infection. As such the work does not provide any data about” correlate of protection from infection” but only analyze the pattern of T cell response after SARS-CoV-2 infection. This should be clearly mentioned.

*Following the Reviewer suggestions, we have accordingly revised the manuscript and all suggestions have been included.*

As such the abstract should be changed. Authors should clarify that the analysis was done after infection. Thus “In this study we aim to evaluate the spike-specific immune responses in patients requiring or not hospitalization for SARS-CoV-2 infection, after breakthrough infection following two doses of BNT162b2 mRNA vaccine”.

*Done.*

2. In addition the authors should also acknowledge that their work substantially confirmed in vaccinated individuals previous work that show lower level of SARS-CoV-2 specific T cells in individuals with severe COVID-19 ( I.e. Tan, A., et al. 2021. Early induction of functional SARS-CoV-2-specific T cells associates with rapid viral clearance and mild disease in COVID-19 patients. Cell Reports, 34(6), p.108728. and Chandran, A. et al. Rapid synchronous type 1 IFN and virus-specific T cell responses characterize first wave non-severe SARS-CoV-2 infections. Cell Reports Medicine 3, 100557–100557 (2022).)

*Following the Reviewer suggestions, we have accordingly revised the manuscript.*

3. The authors should also discuss and pointed out that in severe COVID-19, lymphocytes count is decreasing, and SARS-CoV-2 T cells can be recruited at the site of inflammation and as such the quantity of circulating SARS-CoV-2 T cells can be reduced. This is why it is important to perform longitudinal early analysis ( as in Tan, A., et al. 2021. Early induction of functional SARS-CoV-2-specific T cells associates with rapid viral clearance and mild disease in COVID-19 patients. Cell Reports, 34(6), p.108728. and Chandran, A. et al. Rapid synchronous type 1 IFN and virus-specific T cell responses characterize first wave non-severe SARS-CoV-2 infections. Cell Reports Medicine 3, 100557–100557 (2022) and not only at single time when inflammatory events might be at their peak. This is why is also important to understand when their T cell analysis was performed .

*Following the Reviewer suggestions, we have accordingly revised the manuscript.*

The authors should also acknowledge that the analysis was focused only on Spike, but since samples were collected from donors who were infected with SARS-CoV-2, instead of looking only at Spike-specific T cell responses, it will be complimentary to also look T cells specific for other SARS-CoV-2 structural (Membrane and Nucleoprotein) and non-structural (ORFs) proteins. This will allow the authors to not only better understand the breath of the T cell responses, which is possibly different among the different cohorts, but also know the better representative magnitude of SARS-CoV-2 T cell response, as cellular immunity is contributed by more than Spike-specific T cells. Multi-antigenic T cell response might play a paramount role in preventing severe COVID-19, so it should be discussed in the context of this study.

*We agree with the Reviewer, and we acknowledge her/his concern as limitation of the study. Unfortunately, we do not have samples anymore to provide this adjunctive analysis. In addition, this was out of our study aim and therefore we now suggest that further studies are required at this regard.*

Other minor points:

1. The authors should be clearer about the demographics of the patients recruited, particularly the number of days post-infection, number of days post-vaccination and whether the donors have recovered from COVID-19, as they were not clear on whether they are studying acute or convalescent samples.

*As also required by Reviewer #2, we have included these data in the revised text.*

2. Could the authors clarify on the use of steroid therapy (the statement “since more than 10 days”) or other therapy (i.e. antivirals or antibody), particularly in the “severe/critical COVID-19” cohort.

*As also required by Reviewer #2, we have now specified that none of included patients received steroids before study inclusion*

3. The study could benefit from the availability of clinical parameters describing the patients (degree of peripheral lymphopenia in hospitalized vs non-hospitalized patients, inflammatory markers, etc.)

We agree with the Reviewer, but these data are not available for non-hospitalized (mild-moderate) patients and control (healthy) group. This is now recognized as limitation of the study.

4. It might be better for the authors to include individual datapoints on top of the box plots for the graphs plotted in Figure 1. As it seems like there are obvious outliers, and that cannot be easily inferred with the current graphs. Plotting individual datapoints will allow the readers to better appreciate the data.

*Following the Reviewer’s suggestion, we have not plotted also individual datapoints.*
