# Supplementary material for: Severe and mild-moderate SARS-CoV-2 vaccinated patients show different frequencies of IFNγ-releasing cells: An exploratory study
Source: PLoS One. 2023 Feb 9;18(2):e0281444. doi: 10.1371/journal.pone.0281444 (PMC9910754; doi:10.1371/journal.pone.0281444)
Supplement: Attachment — Submitted filename: Response to Reviewers.docx [file pone.0281444.s002.docx]

**Journal Requirements:**

Please review your reference list to ensure that it is complete and correct. If you have cited papers that have been retracted, please include the rationale for doing so in the manuscript text, or remove these references and replace them with relevant current references. Any changes to the reference list should be mentioned in the rebuttal letter that accompanies your revised manuscript. If you need to cite a retracted article, indicate the article’s retracted status in the References list and also include a citation and full reference for the retraction notice.

*References have been checked and they should be fine per style. To the best of our knowledge, no retracted manuscripts have been included.*

**Additional Editor Comments:**
Manuscript is significantly improved by the authors. However, there are still some minor concerns raised by the reviewer. Please address these concerns and resubmit accordingly.

*Concerns pointed out from Reviewer #2 have been addressed. Please, see below.*

**Reviewers' comments:**

**Reviewer #2:**

Thank you for the opportunity to re-review this manuscript. The authors have made substantial changes and answered all the issues I've raised. The manuscript has substantially improved. Still there are several issues which should be addressed:

- Many minor spelling and grammar mistake should be revised. For example – in the abstract – few spaces are missing, "discriminating" should be discriminate, and so on. This occurs throughout the text.

*Following the Reviewer’s comments, we have checked the entire manuscript and correct.*

- If I understand correctly after the revision – all included patients were diagnosed only upon hospital arrival and not before? If so you should change the sentences describing the first two groups which state "in the previous 10 days". Later you wrote that PCR was positive within 36 hours (results), please state the correct timing in all the places.

*We agree with the Reviewer that the manuscript lacked clarity. The need for a positive PCR swab in the previous 10 days was an inclusion criterion; however, in the results we have specified that all patients resulted positive within 36 hours before inclusion. We have decided to leave only the inclusion timing (36 hours) and to delete the time limit of inclusion criterion. We believe that now the manuscript has improved in its clarity.*

Reviewer #3:

 The authors have addressed my main reservations. The text is more balanced and describes objectively the results obtained.

*Ok, thanks.*
